# Fluorescence Dequenching Makes Haem-Free Soluble Guanylate Cyclase Detectable in Living Cells

**DOI:** 10.1371/journal.pone.0023596

**Published:** 2011-08-17

**Authors:** Linda S. Hoffmann, Peter M. Schmidt, Yvonne Keim, Carsten Hoffmann, Harald H. H. W. Schmidt, Johannes-Peter Stasch

**Affiliations:** 1 Pharma Research Centre, Bayer HealthCare, Wuppertal, Germany; 2 CSIRO Materials, Science and Engineering, Parkville, Victoria, Australia; 3 Julius-Maximilians-University of Wuerzburg, School of Pharmacology and Toxicology, Wuerzburg, Germany; 4 Department of Pharmacology, Maastricht University, Maastricht, The Netherlands; 5 Martin-Luther-University, School of Pharmacy, Halle, Germany; Istituto Dermopatico dell'Immacolata, Italy

## Abstract

In cardiovascular disease, the protective NO/sGC/cGMP signalling-pathway is impaired due to a decreased pool of NO-sensitive haem-containing sGC accompanied by a reciprocal increase in NO-insensitive haem-free sGC. However, no direct method to detect cellular haem-free sGC other than its activation by the new therapeutic class of haem mimetics, such as BAY 58-2667, is available. Here we show that fluorescence dequenching, based on the interaction of the optical active prosthetic haem group and the attached biarsenical fluorophor FlAsH can be used to detect changes in cellular sGC haem status. The partly overlap of the emission spectrum of haem and FlAsH allows energy transfer from the fluorophore to the haem which reduces the intensity of FlAsH fluorescence. Loss of the prosthetic group, e.g. by oxidative stress or by replacement with the haem mimetic BAY 58-2667, prevented the energy transfer resulting in increased fluorescence. Haem loss was corroborated by an observed decrease in NO-induced sGC activity, reduced sGC protein levels, and an increased effect of BAY 58-2667. The use of a haem-free sGC mutant and a biarsenical dye that was not quenched by haem as controls further validated that the increase in fluorescence was due to the loss of the prosthetic haem group. The present approach is based on the cellular expression of an engineered sGC variant limiting is applicability to recombinant expression systems. Nevertheless, it allows to monitor sGC's redox regulation in living cells and future enhancements might be able to extend this approach to in vivo conditions.

## Introduction

The heterodimeric α/β haemprotein soluble guanylate cyclase (sGC) is the physiological receptor for the endogenous gaseous messenger nitric oxide (NO). The prosthetic haem group is non-covalently bound to the β subunit via the haem binding motif Y-x-S-x-R and the axial haem ligand H105 [1], [2]. Binding of NO to the reduced central iron atom of the haem moiety results in an up to 200-fold increase of the conversion rate of guanosine triphosphate (GTP) into the second messenger cyclic guanosine monophosphate (cGMP). cGMP is a key modulator of the cardiovascular system, involved in processes such as smooth-muscle cell relaxation and inhibition of platelet aggregation [3], [4]. Impairment of the NO/sGC/cGMP pathway has been linked to the development of various cardiovascular diseases such as heart failure or arterial hypertension [5].

The prosthetic haem group of sGC has a pivotal role in the activation and stabilisation of the enzyme and its oxidation or removal renders sGC insensitive to NO. Oxidative stress, i.e. the formation of reactive oxygen species (ROS) such as O_2_^−^, has been associated with various cardiovascular diseases [5]. ROS are known to interfere with the NO/sGC/cGMP signalling-pathway via O_2_^−^-mediated scavenging of NO and intermediate peroxynitrite formation, which in turn oxidizes the sGC haem to the NO-insensitive Fe^3+^ state [6], [7], [8]. Furthermore, oxidation of the sGC haem strongly reduces its affinity towards sGC [9], [10] which leads to subsequent loss of the haem [11], [12]. There is a solid body of evidence that sGC is redox regulated and exists in the haem-free form in vivo [11], [13], [14], [15]. In addition, it was shown that haem-free sGC is prone to ubiquitin-mediated degradation [13], [16].

The discovered new NO-independent drug classes, haem-independent sGC activators and haem-dependent sGC stimulators, have been used to distinguish between reduced haem-containing and haem-free sGC. sGC activators such as cinaciguat (BAY 58-2667) mimic the haem group, compete with different porphyrins, and bind in the orphaned sGC haem pocket via the haem anchoring residues Y135 and R139 [9], [13], [17], [18]. This unique mode of action allows this structural class to activate haem-free sGC opening up the possibility of new mechanism-based therapies for those cardiovascular diseases that are associated with oxidative stress [10], [13]. Cinaciguat is currently in clinical development to treat acute decompensated heart failure [11]. In contrast, sGC stimulators like BAY 41-2272 or riociguat (BAY 63-2521) do not activate oxidized or haem-free sGC but show a strong synergism with NO [12].

Despite these therapeutic breakthroughs, the physiological existence of haem-free sGC is a matter of considerable debate due to the lack of a direct method to detect haem-free sGC in living cells. This has left the field with the only option to assay cGMP levels or its functional effects upon exposure to sGC activators when estimating haem-free sGC levels. As a first step to overcome this scientific road block, we aimed to establish a specific and sensitive imaging method based on fluorescence dequenching allowing the direct tracking of the sGC haem status in living cells. As this approach is based on the overexpression of a fluorophore-labelled sGC in a cellular system, its general applicability for in-vivo systems is strongly hampered. Nevertheless, the method allowed for the first time to monitor changes in sGC haem status in living cells under oxidative stress conditions underlining its general usefulness. Future enhancements of the present approach could be based on an engineered sGC-binding a fluorophore like FlAsH, which, in combination with established knock-in techniques, might offer the chance to track changes in the sGC haem status under more physiological conditions.

## Results

### Identification of an appropriate location for the tetracysteine motif

Under native conditions, the presence of the haem group in sGC and its spatial vicinity to the dye are the prerequisites to successfully apply fluorescence dequenching as described in this paper. We assumed that the N-terminus would be in proximity of the haem as observed in the crystal structure of the haem nitric oxide oxygen (H-NOX) domain of *Nostoc sp.* [2].

To identify a position for the tetracysteine (TC, CCPGCC) motif that will not affect the haem binding of sGC, the TC motif was fused either to the N- or C-terminus of sGC or inserted at different positions within the sGC β_1_ coding sequence. Promising intramolecular positions were identified using two approaches. Firstly, we used the molecular model of the sGC haem binding domain as described by Rothkegel *et al.* [19] to identify positions apparently in the proximity of the haem binding pocket. Using this approach, the target positions aa170–175 and aa111–116 were selected for insertion of the TC motif. Secondly, we focused on the region of aa231–310 as previous photoaffinity labelling studies with BAY 58-2667 indicated that this region might be in close proximity to the haem binding pocket [14]. Within this region, we changed positions aa243–248, aa239–244, and aa257–262 into the TC motif. These stretches have been chosen as they already contain a proline similar to the introduced TC motif, CCPGCC. As the introduction of a proline can affect protein structures with higher likelihood than other exchanges this strategy aimed to reduce putative conformational changes to a minimum. The described positions are illustrated in Fig. S1.

**Figure 1:**
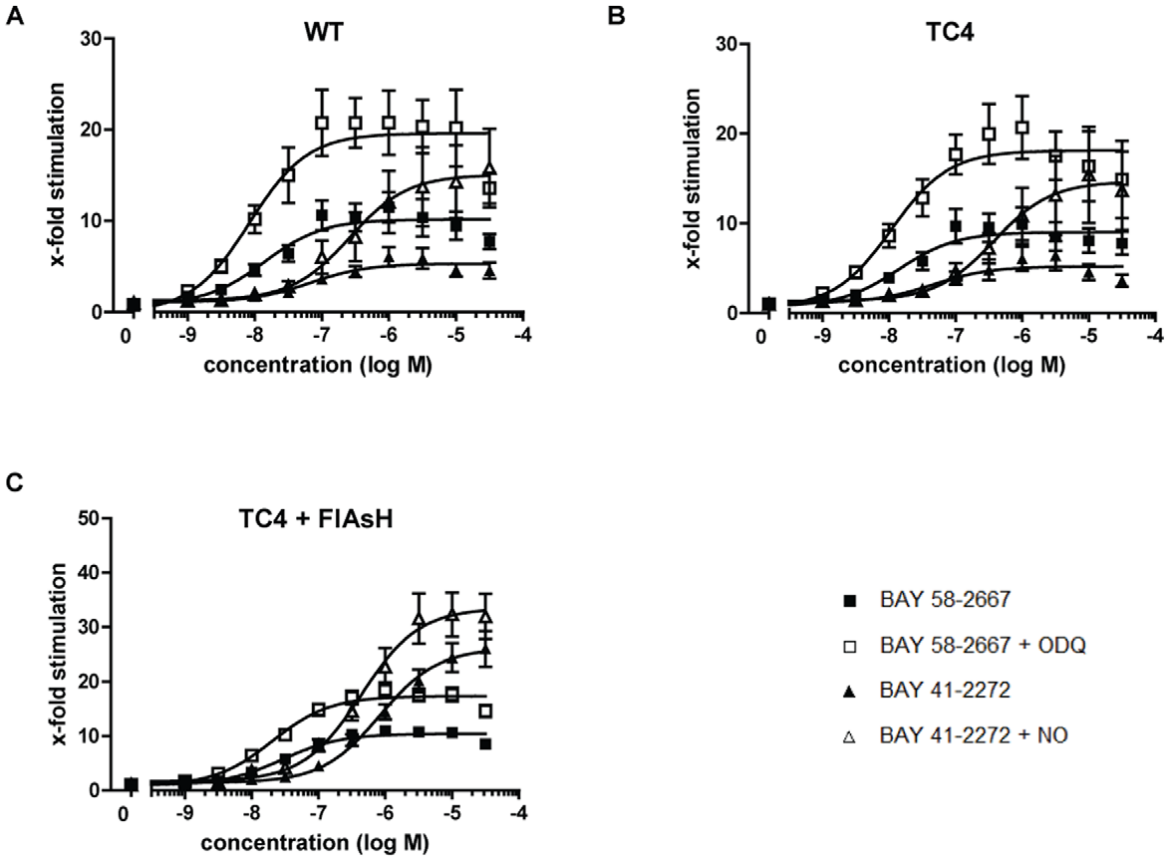
Activation pattern of sGC with tetracysteine (TC) motif. Concentration response curves of WT sGC (A) and sGC with an intramolecular tetracysteine motif without (B) or with FlAsH labelling (C) are shown. cGMP reporter cells were transiently cotransfected with WT α_1_ subunit and WT or a TC motif carrying β_1_ subunit as indicated in the respective figures. Cells were incubated with increasing concentrations of BAY 58-2667 or BAY 41-2272 alone or in combination with 10 µM ODQ or 10 nM DEA/NO (NO), respectively. sGC activity is represented as x-fold stimulation compared to transfected control cells. Data are means ± S.E.M. from 5–14 independent experiments, performed in duplicate. Following basal activities were measured: (A) 10732 relative light units (RLUs), (B) 9720 RLUs, (C) 3541 RLUs.

To verify whether the introduction of the TC motif at different positions had a negative impact on sGC enzyme activity, all TC-β_1_ constructs were screened using the cellular cGMP reporter system described earlier [9], [20]. The dose-response curves of haem-containing WT sGC-expressing cells are shown in Fig. 1A. BAY 58-2667 activated WT sGC under control conditions up to 10-fold. The maximum activity was increased to 20-fold once BAY 58-2667 was combined with the haem-oxidizing sGC inhibitor ODQ. The sGC stimulator BAY 41-2272 stimulated sGC 5-fold and addition of NO increased stimulation to 15-fold.

From all sGC-variants tested, only the TC aa243–248 construct (Fig. 1B) showed an sGC activation pattern very similar to WT sGC. BAY 58-2667 activated the construct 9-fold and this was increased to 18-fold upon addition of ODQ. BAY 41-2272 stimulated the construct 5-fold which was increased to 15-fold by NO. Therefore, this construct was selected for further use and is referred to as TC4 in the following. All other sGC variants showed altered activation profiles compared to the WT enzyme independent whether the TC motif was introduced at the N- or C-terminus or within the sequence of the enzyme (Fig. S2).

To test whether labelling with FlAsH would impact the enzyme activity cGMP read-out was performed as described after cells were labelled with FlAsH (Fig. 1C). BAY 58-2667 activated FlAsH-labeled TC4-WT-sGC up to 11-fold and this was increased to 18-fold upon addition of ODQ. The 26-fold stimulation with BAY 41-2272 was increased to 32-fold when combined with NO. Both results, the synergistic effect of NO and the increase in BAY 58-2667-induced sGC-activity when combined with ODQ indicated the presence of the haem group under native conditions in FlAsH-labelled cGMP-reporter cells and show that neither FlAsH nor the labeling procedure had a negative impact on the enzyme's function.

### Optimization of concentrations of different haem oxidizing compounds

TC4-WT sGC expressing cells were incubated with 100 nM BAY 58-2667 and increasing concentrations of NS 2028, rotenone or ODQ to establish the optimal concentration of the different haem oxidants needed for an efficient oxidation of the sGC haem moiety (Fig. S3). NS 2028 and ODQ have been described to directly oxidize the haem group [21], [22] whereas rotenone is known to increase the intracellular ROS concentration via inhibition of the mitochondrial complex I [23] without having a direct effect on the sGC haem group as NS 2028 or ODQ. BAY 58-2667 activated TC4-WT sGC 6-fold which was increased upon addition of the oxidants. Rotenone and the more effective ODQ derivate NS 2028 were selected for use in further experiments to study the effects of a ROS increasing compound as well as a direct sGC inhibitor.

### Effects of BAY 58-2667, NS 2028 and rotenone on fluorescence of FlAsH-labelled TC4-WT sGC

**Figure 2:**
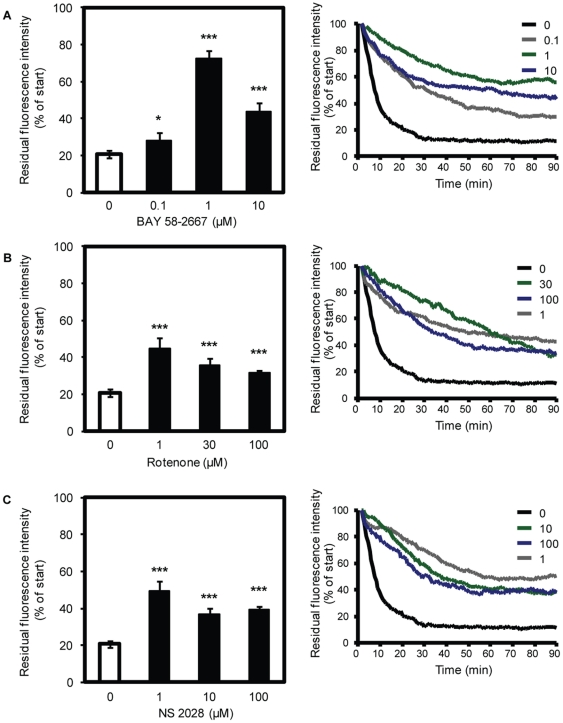
Fluorescence of FlAsH-labelled TC4-WT sGC. cGMP reporter cells were cotransfected with WT α_1_ sGC and TC4-WT β_1_ sGC and labelled with FlAsH. The cells were then incubated with 0.1, 1 or 10 µM BAY 58-2667 (A) 1, 30 or 100 µM rotenone (B) or 1, 10 or 100 µM NS 2028 (C) for 90 min. Fluorescence intensity of single cells was monitored in a time series of 120 laser scans and is expressed as % of start value which was obtained before application of the test compounds. Data are means ± S.E.M. from 9–28 single cells assayed on different days. *p<0.05; ***p<0.005: Student's *t*-test compared to control. Representative traces of the fluorescence measurement as % of start value are given in the right part of the figure.

To test the hypothesis whether loss of the sGC haem group leads to fluorescence dequenching of FlAsH, TC4-WT sGC expressing cGMP reporter cells were incubated for 90 min with BAY 58-2667 which has been shown to replace the native prosthetic group in the haem pocket [9], the sGC oxidant NS 2028, or the ROS-inducing compound rotenone. The latter two compounds lead to oxidation of the haem moiety facilitating the loss of the prosthetic group [8], [24]. Changes in single cell fluorescence were recorded in a time series of 120 laser scans over 90 min. Under control conditions, 90 min of laser-induced bleaching of FlAsH reduced the observed fluorescence to 21±2% (Fig. 2). Incubation of FlAsH-labelled cells with different concentrations of BAY 58-2667 increased the residual FlAsH-fluorescence to a maximum of 72±4% compared to control (Fig. 2A). Next we examined whether treatment with the haem oxidants and subsequent loss of the sGC haem group dequenched FlAsH fluorescence and resulted in higher fluorescence compared to control. Treatment of cells with 1, 30 and 100 µM rotenone for 90 min led to an significant increase of residual fluorescence intensity to 46±8% (*p*<0.005), 32±4% (*p*, 0.005) and 30±4% (*p*<0.005), respectively, when compared to control (Fig. 2B). Treatment of cells with NS 2028 resulted in significant higher fluorescence intensity compared to control as well (Fig. 2C). This effect was significant for 1, 10 and 100 µM NS 2028 which increased fluorescence intensity to 49±5% (*p*<0.005), 37±4% (*p*<0.005) and 35±5% (*p*<0.005), respectively. These data suggest the replacement of the haem group by BAY 58-2667 or oxidation-induced haem loss induced by rotenone or NS 2028. Representative traces of the measurement of fluorescence are shown in Fig. 2. To measure the fluorescence intensity single cells were marked with “regions of interest” (ROI) over which fluorescence was integrated. ROIs over non-transfected cells and without cells were used to measure background levels and subtracted from the single cell values. The decrease in fluorescence observed after 90 min is due to bleaching effects which are the same in treated and untreated cells. Therefore, comparison of the residual fluorescence intensity in treated and untreated cells shows fluorescence dequenching.

### Effects of NS 2028 on FlAsH-labelled TC4-haem-free sGC and ReAsH-labelled TC4-WT sGC

To validate that the observed increases in fluorescence were indeed based on dequenching, two controls were used. First, the haem-free mutant TC4-Y135A/R139A, was used to exclude that any increases in fluorescence were due to unknown or artificial, i.e. haem-independent mechanisms. For that purpose, TC4-Y135A/R139A sGC expressing cells were incubated with 100 µM NS 2028 for 90 min. In contrast to TC4-WT sGC, the fluorescence was only slightly and not significantly increased (Table 1). Second, we used ReAsH-labelled TC4-WT sGC. As shown in Fig. S4, the emission spectrum of ReAsH overlaps only slightly with the absorption spectrum of haem-containing sGC. We therefore assumed that the sGC haem would quench ReAsH to a much lesser extent than observed for FlAsH. Again, treating the cells with 100 µM NS 2028 for 90 min did not affect residual fluorescence intensity of ReAsH (Table 1) indicating that the observed increase for FlAsH fluorescence has been indeed due to haem loss.

**Table 1:** Effects of 100 µM NS 2028 on fluorescence intensity of FlAsH labelled haem- free TC4-Y135A/R139A sGC (A) and ReAsH labelled TC4-WT sGC (B) after 90 min incubation.

	TC4-Y135A/R139A sGC	TC4-WT sGC
	FlAsH	ReAsH
Control	12.69±1.16	65.99±7.16
100 µM NS 2028	16.56±3.36	73.26±11.53

cGMP reporter cells were cotransfected with WT α_1_ sGC and the respective β_1_ sGC and labelled with FlAsH or ReAsH. Fluorescence intensity of single cells was monitored in a time series of 120 laser scans and is expressed as % of start value which was obtained before application of the test compounds. Data are means ± S.E.M. from 3–5 single cells assayed on different days.

### Influence of NS 2028 and rotenone pre-treatment on activity of TC4-WT sGC

**Figure 3:**
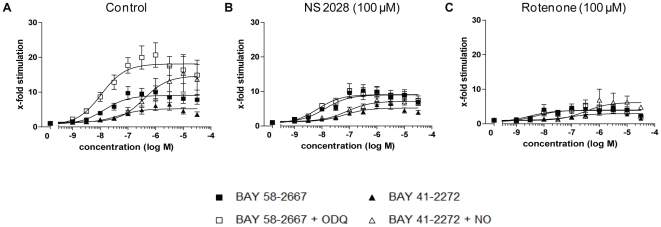
Effect of 90 min incubation with NS 2028 and rotenone on sGC activity. cGMP reporter cells expressing TC4-WT sGC were pre-incubated with 100 µM NS 2028 or rotenone and then incubated with increasing concentrations of BAY 58-2667, BAY 41-2272 alone or in combination with 10 µM ODQ or 10 nM DEA/NO (NO), respectively. Enzyme activity is expressed as x-fold stimulation compared to pre-treated but not stimulated control. Data are means ± S.E.M. from 3–10 independent experiments, performed in duplicate. A basal activity of 7747 relative light units was measured. A) is identical to Fig. 1B.

To test if treatment with haem oxidants affects the haem status of TC4-WT sGC as implicated by increased FlAsH fluorescence, its activity was measured 90 min after pre-treatment with 100 µM NS 2028 or rotenone (Fig. 3). This diminished BAY 41-2272-induced cGMP levels and abolished the effects of ODQ on BAY 58-2667-induced sGC activity as well as the synergistic effect of NO on BAY 41-2272 induced sGC stimulation. Similar results were obtained for WT-sGC expressing cells after pre-treatment with NS 2028 or rotenone (Fig. S5). These data indicated that both, the haem oxidant NS 2028, and rotenone, indeed affected the oxidation state of the haem group.

### Effects of NS 2028 and rotenone on TC4-WT sGC protein levels

Haem oxidation leads also to sGC degradation [13], [15], [17], [25]. Thus the effects of 90 min treatment with 100 µM NS 2028 or rotenone on sGC protein levels were tested in TC4-WT sGC expressing cells (Table 2). Although NS 2028 did not affect α_1_ sGC protein levels after 90 min treatment, the haem-binding β_1_ subunit of sGC was significantly reduced to 78±17% of control (*p*<0.005). 90 min treatment with rotenone significantly decreased protein levels of both sGC α_1_ and β_1_ (65±10 and 81±11%, respectively; *p*<0.005). Incubating WT sGC expressing cells under the same conditions produced similar results (Table S1). In contrast, when TC4-Y135A/R139A expressing cells were incubated with 100 µM NS 2028 for 90 min no change in sGC protein levels was observed (data nor shown).

**Table 2:** Effects of 90 min pre-treatment with 100 µM NS 2028 or rotenone on TC4-WT sGC protein levels.

	α_1_ sGC	β_1_ sGC
Control	100	100
100 µM NS 2028	99.1±4.75	84.52±15.07***
100 µM Rotenone	65.01±9.65***	80.57±10.81***

cGMP reporter cells were transiently cotransfected with the WT α1 subunit and the TC4-WT β_1_ subunit. α_1_ sGC and TC4-WT β_1_ sGC were detected separately and protein levels were measured by densitometric analysis. sGC protein levels were normalized to the respective control which was set as 100%. Data are means ± S.E.M. from 3–9 independent experiments.

***p<0.005: Student's *t*-test.

## Discussion

We here show that it is possible to directly monitor the sGC haem oxidation state in intact cells. By inserting the TC motif into the coding sequence of the β_1_ subunit of sGC in transiently transfected Chinese hamster ovary cells, we were able to monitor sGC fluorescence intensity upon addition of the haem mimetic BAY 58-2667, the haem oxidant, NS 2028, and the ROS generating compound, rotenone. The test compounds lead to an increase of FlAsH fluorescence intensity when compared to control. This was neither observed in the ReAsH-labelled negative control nor when a haem-free sGC mutant was expressed and labelled with FlAsH. These direct fluorescence measurements were accompanied by indirect probing of sGC activity via the established pharmacological tools BAY 58-2667 and BAY 41-2272 and by monitoring sGC stability as another marker for haem loss.

The NO/sGC/cGMP pathway has been shown to be impaired in various cardiovascular diseases mainly by a reduced bioavailability of NO and, in parallel, by reducing the sensitivity of sGC towards its agonist NO [5], [7], [25], [26], [27]. Oxidation or loss of the sGC prosthetic haem group may be the cause for this reduced sensitivity as oxidized or haem-free sGC is unresponsive to NO [11], [13]. There is compelling evidence that haem-free sGC exists under physiological conditions and that this pool is increased in pathophysiological situations associated with the increased production of ROS [13], [24]. Very recently it was shown for *Manduca sexta* sGC that haem oxidation leads to haem loss because the ferric state is unstable. In contrast, the ferrous state is highly stable and resistant to haem loss [8]. Furthermore, haem-free sGC is prone to ubiquitin-mediated degradation and this might be at least partly the cause for the observed decreased sGC protein levels in animal models of cardiovascular diseases [13], [16], [25], [28].

Although imaging methods employing FRET and non-FRET methods have extensively been used to monitor intracellular cGMP dynamics [29] no such method is available to investigate the haem content of sGC. Therefore, the intracellular existence of haem-free sGC in living cells and its regulation is still a matter of debate.

To investigate conformational changes of proteins several studies used the ability of the haem group to quench the fluorescence of other chromophores if the spectra of haem and the respective chromophore overlap. Kosarikov and coworkers [30] used the intrinsic fluorescence of tryptophans to investigate conformational changes of sGC. They observed that the intrinsic fluorescence of sGC decreased upon addition of NO and suggested that the intrinsic tryptophan fluorescence is quenched to some extend by sGC's haem group due to NO-induced conformational changes or the shift of the Soret peak of the nitrosyl-haem complex. In cytochromes fused to green fluorescent protein (GFP) the fluorescence of GFP was quenched when the apo-enzymes were reconstituted with haemin [31]. Another study using fluoresceinisothiocyanat (FITC) labelled cytochrome P450scc showed that the fluorescence of FITC is effectively quenched in the presence of the haem and even changes in the redox state of the protein could be detected [32]. The ability of the haem to quench the fluorescence of other chromophores thereby provides a powerful tool to examine the haem content of proteins. As the use of the intrinsic tryptophan fluorescence is only applicable to purified proteins, sGC had to be specifically labelled with an extrinsic fluorophor that can be used as read-out to estimate the sGC haem content. We decided against fluorescent fusion proteins such as GFP as these proteins are bulky and likely to sterically interfere with sGC folding. Other fluorophors such as FITC, which represent very powerful tools when used to label purified proteins, react with reactive groups on any protein ruling out the possibility to specifically label sGC in living cells.

Conversely, the biarsenical dyes FlAsH and ReAsH, which we used, allow for the specific labelling of a single protein within cells [33], [34], [35]. These dyes have the additional advantage that they are rather small in size (<700 Da compared to 27 kDa of GFP) [36], cell permeable, and bind tightly to a small tetracysteine (TC; CCPGCC) motif that can be readily introduced by mutagenesis [33], [34]. One of the major advantages of these dyes is that they remain non-fluorescent when complexed to 1,2-ethanedithiol but increase their fluorescence 50,000-fold once bound to their target protein [34]. The combination of these beneficial characteristics has resulted in the use of FlAsH and ReAsH for a broad range of fluorescence-based applications [37], [38], [39], [40]. An excellent example for the application of biarsenical dyes is the use of FlAsH in dynamic FRET studies with cyan fluorescent protein (CFP) [35], [41]. Employing FlAsH instead of the classically used YFP in the FRET pair CFP/YFP made it possible to measure G-protein coupled receptor activation in living cells without altering the receptor function which was a major drawback of the use of YFP.

**Figure 4:**
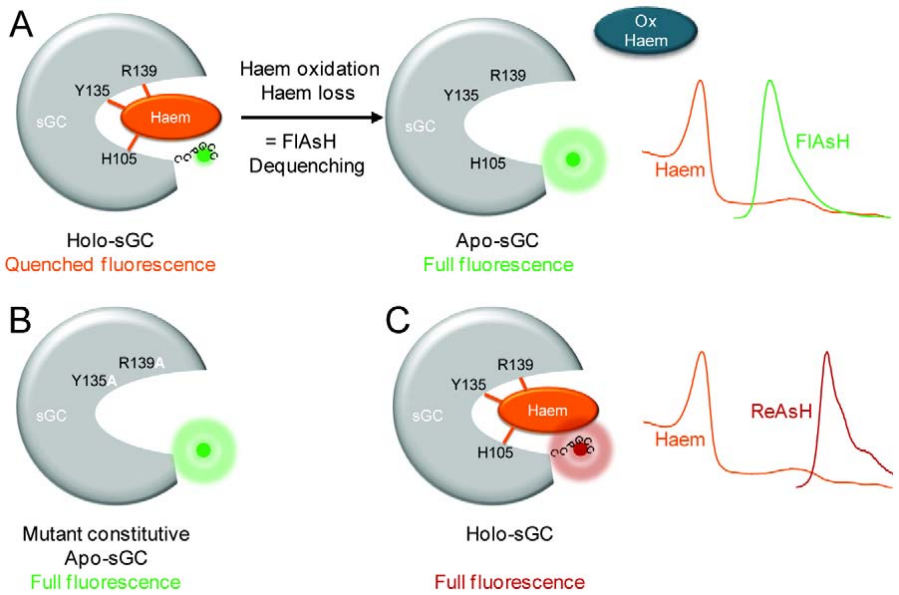
Principle of the fluorescence dequenching method. A) The green fluorescent biarsenical dye FlAsH binds to the tetracysteine motif CCPGCC in sGC. In holo-sGC, the fluorescence of FlAsH is quenched by the haem group as energy from FlAsH can be transferred to the haem group due to the overlap in the respective absorption and emission spectra. Haem oxidation leads to loss of the heam group and dequenching of FlAsH fluorescence. Hence, apo-sGC shows full FlAsH fluorescence. B) Replacement of the haem anchoring residues Y135 and R139 with alanine results in a constitutive haem-free sGC. As FlAsH fluorescence cannot be quenched by the haem group FlAsH shows full fluorescence. This sGC form was therefore used as a negative control to test if changes in FlAsH fluorescence are actually due to haem loss. C) Like FlAsH, the red fluorescent biarsenical dye ReAsH binds to the CCPGCC motif. But compared to FlAsH the emission spectrum of ReAsH overlaps with the absorption spectrum of the haem group to a much lesser extent. Consequently, the haem group does not quench the ReAsH fluorescence. ReAsH could therefore be used as a negative control to test if changes in FlAsH fluorescence are due to the overlapping spectra of haem and FlAsH.

The most suitable biarsenic dye to track the presence of the sGC haem group via fluorescence-dequenching would have been CHOxAsH [34] as its emission spectrum shows good overlap with the sGC Soret peak. This characteristic would have ensured an efficient energy transfer and, as a consequence, robust fluorescence dequenching. However, CHOxAsH (generously provided by Roger Tsien) showed limited photostability and brightness compared to FlAsH or ReAsH [34] preventing its use in our study. Hence we used the green dye FlAsH to establish our approach. Although the emission spectrum of FlAsH does not overlap with the Soret peak, quenching of FlAsH fluorescence by the haem group was anticipated due to the overlap of the α and β absorbance bands of the haem with the emission spectrum of FlAsH. Previously it was demonstrated that spectral overlaps similar to sGC haem and FlAsH result in effective energy transfer [42], [43], [44]. As haem oxidation is assumed to render sGC haem-deficient treatment with haem oxidants should result in an increased fluorescence of FlAsH. The basic principle of the method employed in our study is illustrated in Fig. 4A.

A prerequisite to establish a reliable fluorescence dequenching-method is the presence of the sGC haem group under native conditions. From all sGC variants tested, only the TC4-WT sGC showed an activation and stability pattern similar to WT sGC, all further experiments were conducted with this sGC construct. In order to validate our test system, haem loss of sGC was induced by oxidizing the prosthetic group of the enzyme by two different mechanisms: Direct sGC haem oxidation via ODQ/NS 2028 [21], [22] and, alternatively, by incubation with rotenone, which is known to increase cellular ROS levels by the inhibition of the mitochondrial complex I [23]. With respect to direct haem oxidation, NS2028 was more effective than ODQ (Fig. S3) and therefore used for all further experiments. The effectiveness of NS 2028 and rotenone to induce sGC haem loss was pharmacologically confirmed by sGC activity assays (Fig. 3).

A 90 min incubation with BAY 58-2667, NS 2028 or rotenone resulted in a significant increase of residual fluorescence intensity compared to the untreated control. These results suggested that replacement of the native prosthetic group with BAY 58-2667 and haem loss following haem oxidation result in dequenching of FlAsH fluorescence. As haem-free sGC has been published to be prone to proteasomal degradation [13], [16] we analysed protein levels of oxidant-pre-treated TC4-WT sGC expressing cells to validate that incubation with NS 2028 or rotenone results ultimately in haem loss (Table 2).

Whereas rotenone led to a decrease of both sGC subunits, treatment with NS 2028 decreased only the expression levels of the β_1_ subunits. This reduction after 90 min was less pronounced than observed in earlier studies using 24 h incubations [13], [16], [17], [25]. BAY 58-2667-induced sGC activity was reduced after 90 min incubation with NS 2028 or rotenone, mirroring the trend of the reduced sGC protein levels. This is in agreement with previous work, which demonstrated that reduced sGC protein levels result in diminished sGC activity [17]. Proteasomal degradation might also be an explanation for the reduced fluorescence in cells treated with more than 1 µM of the oxidants as in cells treated with 1 µM,

Taken together sGC activity measurements and the observed oxidation-induced sGC degradation by NS 2028 and rotenone indicated that treatment with these drugs results in increased amounts of cellular haem-free sGC. Furthermore, it is well established that BAY 58-2667 competes with the haem group for its binding sites and can replace the native group from the enzyme [9]. Therefore we assumed that incubation of FlAsH-labelled cells with BAY 58-2667 might lead to an increase of FlAsH fluorescence when the native group is replaced. This was clearly demonstrated by measurements of FlAsH fluorescence and is in line with the finding when NS 2028 and rotenone were used. Therefore it is reasonable to assume that the observed increase in residual fluorescence intensity is therefore indeed due to a loss of the haem group. To further strengthen these findings we validated the fluorescence dequenching-method by expressing a haem-free variant of TC4 sGC as well as the use of ReAsH (Fig. 4B and C). The double mutation Y135A/R139A was introduced into TC4-WT sGC as this mutation has been shown to result in the expression of haem-free sGC, which, in contrast to the haem free mutant H105F, cannot be reconstituted with porphyrins [9]. With the use of this haem-free mutant we tested if the observed effects on residual fluorescence intensity are caused by a loss of haem. Indeed, residual fluorescence intensity of TC4-Y135A/R139A sGC was unaffected by 90 min incubation with 100 µM NS 2028. At this point it is worth mentioning, that the increased fluorescence intensity compared to control observed with TC4-WT sGC might also result from a change in spectral properties of the haem still bound to sGC or of the released haem. Currently studies using recombinant purified TC4-WT sGC and haem-free variants are underway to rule out this possibility. In addition, the results of the activity measurements and the reduced sGC protein levels argue against this possibility. To verify that the observed fluorescence dequenching relies on the overlapping spectra of FlAsH and haem we used ReAsH as an alternative dye. The emission spectrum of this biarsenical dye only very slightly overlaps with the haem spectrum and therefore no dequenching effect should be observed upon oxidation-induced haem removal. Again, 100 µM NS 2028 were tested and 90 min incubation with the compound did not affect residual fluorescence intensity of ReAsH. Bleaching of ReAsH was less compared to FlAsH. This is in agreement with previous studies, which showed that red fluorescence is more resistant to photobleaching than green fluorescence [45]. Our data with TC4-Y135A/R139A sGC and ReAsH thus indicate that the observed increased residual FlAsH fluorescence upon treatment with oxidants is very likely due to the loss of the haem group.

As the fluorescence dequenching method is a new developed approach to determine the haem status of sGC and as such still in its infancy, limitations of the method should not remain unmentioned here. A major drawback of the results presented here is that although significant changes in FlAsH fluorescence compared to control could be detected no clear concentration dependency of fluorescence dequenching could be observed neither for BAY 58-2667, NS 2028 nor rotenone. This might be due to unknown effects of the oxidizing compounds or unbound haem in the complex surrounding of the intact cell. Nevertheless, the results demonstrate the sGC haem loss and the feasibility of this new approach in general. Further research is warranted to extend the findings and method to other cell lines beyond Chinese hamster ovary cells and by increasing its sensitivity.

Despite these limitations of the study we could demonstrate that the fluorescence dequenching-method provides the first experimental approach to directly measure the presence of haem-free sGC in intact cells and might be applied to gain new insights into the redox regulation of sGC and its targeting by haem mimetics such as BAY 58-2667. Future improvements of the presented approach could be based on a fluorophore-binding sGC, which, in combination with established knock-in techniques, might offer the chance to track changes in the sGC haem status under more physiological conditions and might even allow to investigate the influence of different disease states on the redox equilibrium of sGC.

## Materials and Methods

### Materials

BAY 58-2667 (4-(((4-carboxybutyl){2-((4-phenethylbenzyl)oxy) phenethyl}amino)methyl-(benzoic)acid) and BAY 41-2272 (5-cyclopropyl-2-(1-(2-fluoro-benzyl)-1*H*-pyrazolo(3,4-*b*)pyridin-3-yl)-pyrimidin-4-ylamine) were synthesized as described [46], [14]. ODQ (1H-(1,2,4)-oxadiazole(4,3-*a*)quinoxalin-1-one) was purchased from Tocris Bioscience (Avonmouth, UK) and NS 2028 (4*H*-8-Bromo-1,2,4-oxadiazolo(3,4-d)benz(b)(1,4)oxazin-1-one) was purchased from Axxora GmbH (Loerrach, Germany). FlAsH and ReAsH were purchased as LumioGreen and LumioRed, respectively, from Invitrogen (Carlsbad, CA, USA). All other chemicals were of analytical grade and obtained from Sigma (Steinheim, Germany). The generation of the cGMP reporter cell line was described recently [20].

### Mutagenesis and tagging of β_1_ sGC

The biarsenical dyes fluorescein arsenical helix binder (FlAsH) and resorufin arsenical helix binder (ReAsH) bind tightly to a small tetracysteine (TC) motif that consists of the amino acid sequence CCPGCC and can be readily introduced in different proteins [33], [34]. Mutagenesis was performed using QuikChange® site-directed mutagenesis (Stratagene, La Jolla, CA) according to the manufacturer's protocol. The sequences of the primers used to introduce the desired mutations are shown in Table S2. The accuracy of the mutations was verified by sequencing (Invitek, Berlin, Germany). C-terminal and N-terminal tagging of WT β_1_ sGC was achieved by cloning the coding sequence for the WT β_1_ subunit in the Gateway® expression vectors pcDNA6.2/cLUMIO-DEST and pcDNA6.2/nLUMIO-DEST (Invitrogen, Carlsbad, CA, USA). In parallel, the vector pRC/CMV, which has been commonly used in our lab to express sGC in mammalian cell lines [9], [20], was amended by the N-terminal addition of the TC-motif followed by a linker sequence.

### Transient transfection

Transient cotransfection of the α_1_ and β_1_ subunit was performed as described elsewhere [9]. For sGC activity measurements, 10^4^ cGMP reporter cells (see below) per well were seeded on 96-well microtiter plates and cultured for 1 day at 37°C, 5% CO_2_. Cells were then cotransfected by adding 19 ng α_1_- and 19 ng β_1_-plasmid (as 1 µg/µl in ultrapure H_2_O) in a mixture of 0.13 µl Plus™Reagent and 0.8 µl of Lipofectamine™ (Invitrogen, Carlsbad, USA) to 100 µl Opti-MEM®I Reduced Serum Medium (Invitrogen) per well for 3 h. Subsequently, the medium was exchanged against serum-containing medium and cells were incubated for 24 h. cGMP read-outs were performed as described before [20].

Cells used for fluorescence intensity measurement were seeded on LabTek™II chambered coverglasses (Nunc, Roskilde, Denmark) at a density of 2×10^4^ cells per well and cultured as described above. Transfection of adhered cells was achieved by applying a transfection mixture containing 0.5 µg α_1_- and 0.5 µg β_1_-plasmid, 58 µl Opti-MEM®I and 0.5 µl Lipofectamine™2000 (Invitrogen) diluted in 58 µl Opti-MEM®I per well according to the manufacturer's instructions. After 4 h 100 µl serum-containing medium was added and cells were propagated for further 24 h before labelling expressed sGC with FlAsH or ReAsH.

### Labelling with FlAsH and ReAsH

Labelling of transfected cells grown on cover glasses with FlAsH or ReAsH was performed as described [41], [47]. To ensure complete complexation of the biarsenical dyes with 1,2-ethanedithiol (EDT) the dye was pre-incubated with 25 mM EDT. Cells were then washed three times with Hank's buffered salt solution (HBSS without phenol red, Invitrogen) containing 1 g/l glucose. Subsequently, cells were incubated with the diluted FlAsH/EDT mixture (final concentration: 0.5 µM FlAsH; 12.5 µM EDT) for 1 h at room temperature. Afterwards, cells were washed three times with 250 µM EDT in HBSS to reduce non-specific labelling. Finally, before measuring fluorescence intensity, cells were washed three times with HBSS to remove excess EDT.

### Measurement of residual fluorescene intensity

To determine whether removal of the sGC haem group results in an increased fluorescence of FlAsH or ReAsH, the fluorescence intensity of single cells was measured with a Zeiss Confocal Microscope LSM 510 (Carl Zeiss, Jena, Germany). FlAsH was excited at 488 nm and detected at 505 nm to 545 nm (using the main beamsplitter for 488/543 nm and a secondary beamsplitter for 545 nm in combination with a long pass filter of 505 nm). ReAsH was excited at 543 nm and detected using the main beamsplitter for 488/543 nm and a long path filter of 560 nm. The same settings for contrast and noise suppression were used in all measurements using an oil Plan 63× objective (Carl Zeiss, Jena, Germany). Fluorescence intensity was measured in a time series consisting of 120 laser scans distributed equally over the incubation time of 90 min. The mean value of the first three scans was used as a start value to calculate residual fluorescence intensity after the respective incubation time. Before the fourth scan the oxidant (NS 2028 or rotenone) or BAY 58-2667 was added and incubated for the remaining time. Because of bleaching effects fluorescence intensity of the analyzed cells decreased over the time series. As the spectra of FlAsH and haem overlap (Fig. S4), we hypothesized that FlAsH fluorescence would be quenched by the prosthetic group under control conditions, but oxidation of the haem moiety followed by its subsequent loss would result in an increase of FlAsH fluorescence. As the spectra of ReAsH and haem do not overlap as observed with Flash, the red-fluorescing dye could be used as an important negative control to validate the overall experimental approach. Images were taken with an integrated charge-coupled device camera, processed, and analyzed with the LSM Image software (Carl Zeiss). The starting fluorescence of treated and control cells was set as 100 and residual fluorescence intensity was expressed as a percentage of this value.

### cGMP reporter cell line and cGMP read-out

cGMP reporter cells were generated and cultured as previously described [9], [20]. Briefly, the cGMP reporter cells consist of Chinese hamster ovary cells stably transfected with the cGMP gated Ca^2+^ channel CNG2 and aequorin, which translates increasing levels of intracellular Ca^2+^ into bioluminescence. In addition, these cells were transiently transfected with the WT α_1_ subunit and the TC motif-containing β_1_ subunit of sGC as described above. Cells were pre-treated with NS 2028 or rotenone as indicated below. To determine the sGC activation profile, cells were incubated for 10 min at room temperature with increasing concentrations of the sGC stimulator, BAY 41-2272, or sGC activator, BAY 58-2667, both in the absence or presence of 10 nM DEA/NO and 10 µM ODQ, respectively. 3-Isobutyl-1-methylxanthin (0.2 mM) was added to all buffers to prevent cGMP degradation by endogenous phosphodiesterases. The bioluminescence was initiated by addition of 10 mM CaCl_2_ and correlates directly with the intracellular cGMP content [20]. Bioluminescence was measured in relative light units (RLU). Data shown in Figures 1 and S 2 were obtained in independent sets of experiments using different batches of cells.

### Western blotting

Cells were seeded in 6-well plates, grown to confluence and then incubated with 100 µM NS 2028 or 100 µM rotenone for 90 min. Cells were then harvested and lysed to extract protein as described before [48]. 30 µg of total protein were separated by SDS-PAGE and blotted onto nitrocellulose membranes. Using polyclonal antibodies directed against specific epitopes of the sGC α_1_ (Sigma, Steinheim, Germany) and β_1_ subunit (Cayman Chemical Company, Ann Arbor, Michigan, USA) the individual sGC subunits were detected separately. Actin was used as loading control and was detected with commercially available antibodies (Sigma, Steinheim, Germany). Detection was performed by the ECL method (Amersham/GE Healthcare, Buckinghamshire, UK). Protein levels were determined by densitometric analysis of the specific protein bands (GS-800 Calibrated Densitometer, Quantity One Analysis Software, BioRad, Munich, Germany). Values were normalized to the respective untreated control, which was set to 100% as well as to the respective actin ratio.

### Statistics

Data are presented as means ± S.E.M of n experiments. GraphPad Prism software version 4.02 (GraphPad Software Inc., San Diego, CA, USA) was used for curve fitting. Statistical comparisons were performed using the paired Student's *t*-test.

## Supporting Information

Figure S1 **Overview of the tetracysteine (TC) motifs introduced into the primary sequence of β_1_ sGC.** The haem-binding motif is highlighted in red, the stretch of amino acids identified by photoaffinity labelling [14] is shaded in grey, and the regions in which the TC motif has been introduced are marked in green.
(TIF)

Figure S2 **Activation pattern of WT sGC (A), sGC with an intramolecular tetracysteine motif (TC; B–E) and TC tagged sGC (F–H).** The positions of the intramolecular TC motifs are shown above each graph. The TC was tagged N-terminally by site-directed mutagenesis (F) or by cloning WT β_1_ sGC into pcDNA6.2/cLUMIO-DEST vector (G). N-terminal tagging was achieved by cloning WT β_1_ sGC into pcDNA6.2/nLUMIO-DEST vector (H). WT α_1_ sGC and the respective β_1_ subunit were cotransfected into cGMP reporter cells. Cells were incubated with increasing concentrations of BAY 58-2667, BAY 41-2272 alone or in combination with 10 µM ODQ or 10 nM DEA/NO (NO), respectively. sGC activity is represented as x-fold stimulation compared to transfected but unstimulated control. Data are means ± S.E.M. from 2–11 independent experiments, performed in duplicate. Following basal activities were measured: (A) 10732 relative light units (RLUs), (B) 355 RLUs, (C) 570 RLUs, (D) 23318 RLUs; (E) 6518 RLUs, (F) 1896 RLUs, (G) 15741 RLUs, (H) 19822 RLUs.
(TIF)

Figure S3 **Effects of NS 2028, rotenone and ODQ on BAY 58-2667-induced TC4-WT sGC activity.** cGMP reporter cells were transiently cotransfected with WT α_1_ sGC and TC4-WT β_1_ sGC and incubated with 100 nM BAY 58-2667 and increasing concentrations of NS 2028, rotenone and ODQ. sGC activity is represented as x-fold stimulation compared to transfected but non-stimulated control. Data are means ± S.E.M. from 7–18 independent experiments, performed in duplicate. Basal sGC activity resulted in 11218 RLUs.
(TIF)

Figure S4 **Spectra of the biarsenical dyes FlAsH and ReAsH and of Fe^2+^-haem-containing sGC.** Data for relative fluorescence intensity of FlAsH and ReAsh was adopted from the website of Roger Tsien's lab (www.tsienlab.ucsd.edu/Documents). Absorbance spectrum of reduced sGC was adopted from [9].
(TIF)

Figure S5 **Activation pattern of WT sGC expressing cGMP reporter cells.** Cells were incubated with increasing concentrations of BAY 58-2667, BAY 41-2272 alone or in combination with 10 µM ODQ or 10 nM DEA/NO (NO), respectively.
(TIF)

Table S1 **Effects of 90 min pre-treatment with 100 µM NS 2028 or rotenone on WT sGC protein levels.** cGMP reporter cells were transiently cotransfected with the WT α_1_ and β_1_ subunit of sGC. α_1_ sGC and WT β_1_ sGC were detected separately and protein levels were measured by densitometric measurement. sGC protein levels were normalized to the respective control which was set as 100%. Data are means ± S.E.M. from 4–6 independent experiments. **p<0.01; ***p<0.005: Student's *t*-test.
(DOC)

Table S2 **Primers used to insert the tetracysteine motif or the Y135A/R139A mutation at the desired positions.**
(DOC)
