# Supplementary figures and images for: Fluorescence Dequenching Makes Haem-Free Soluble Guanylate Cyclase Detectable in Living Cells
Source: PLoS One. 2011 Aug 17;6(8):e23596. doi: 10.1371/journal.pone.0023596 (PMC3157391; doi:10.1371/journal.pone.0023596)

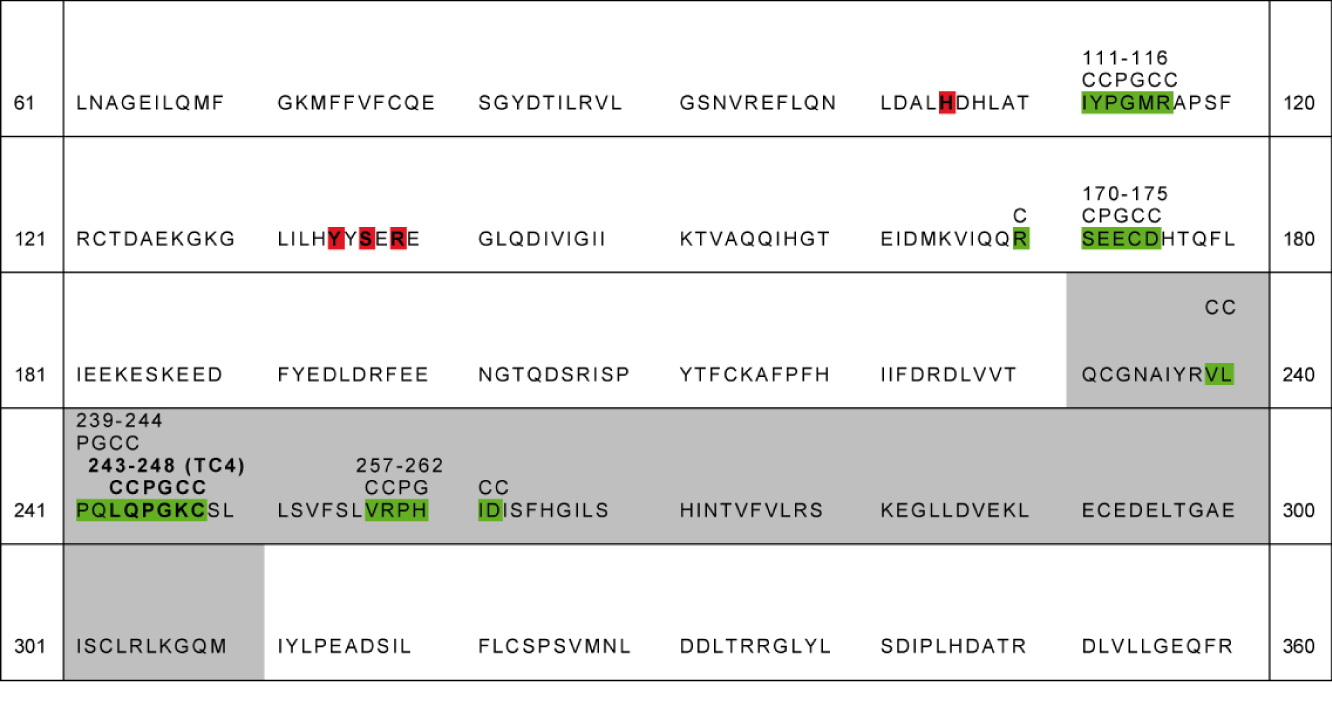

Supplement: Figure S1 — Overview of the tetracysteine (TC) motifs introduced into the primary sequence of β1 sGC. The haem-binding motif is highlighted in red, the stretch of amino acids identified by photoaffinity labelling [14] is shaded in grey, and the regions in which the TC motif has been introduced are marked in green. (TIF) [file pone.0023596.s001.tif]

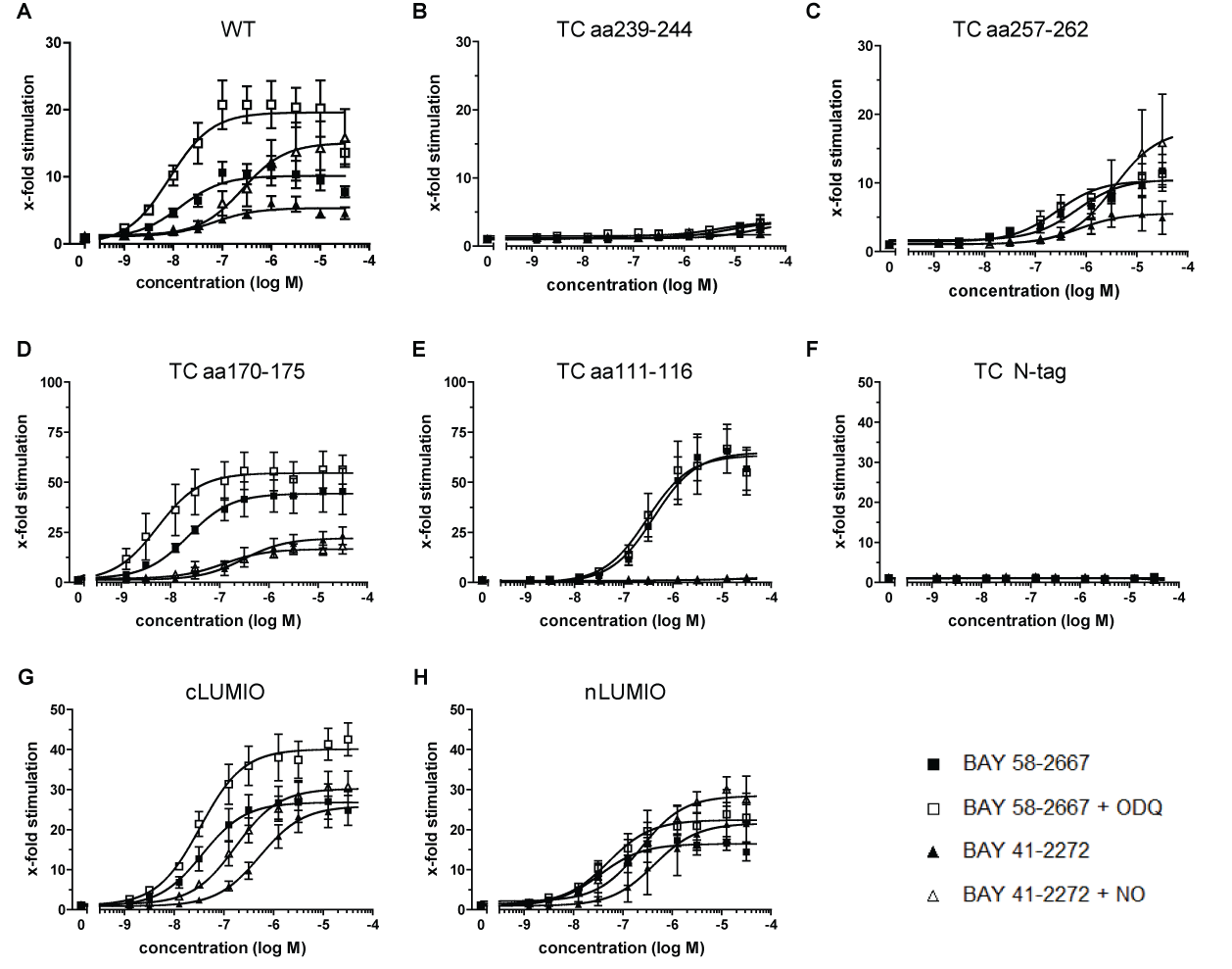

Supplement: Figure S2 — Activation pattern of WT sGC (A), sGC with an intramolecular tetracysteine motif (TC; B–E) and TC tagged sGC (F–H). The positions of the intramolecular TC motifs are shown above each graph. The TC was tagged N-terminally by site-directed mutagenesis (F) or by cloning WT β1 sGC into pcDNA6.2/cLUMIO-DEST vector (G). N-terminal tagging was achieved by cloning WT β1 sGC into pcDNA6.2/nLUMIO-DEST vector (H). WT α1 sGC and the respective β1 subunit were cotransfected into cGMP reporter cells. Cells were incubated with increasing concentrations of BAY 58-2667, BAY 41-2272 alone or in combination with 10 µM ODQ or 10 nM DEA/NO (NO), respectively. sGC activity is represented as x-fold stimulation compared to transfected but unstimulated control. Data are means ± S.E.M. from 2–11 independent experiments, performed in duplicate. Following basal activities were measured: (A) 10732 relative light units (RLUs), (B) 355 RLUs, (C) 570 RLUs, (D) 23318 RLUs; (E) 6518 RLUs, (F) 1896 RLUs, (G) 15741 RLUs, (H) 19822 RLUs. (TIF) [file pone.0023596.s002.tif]

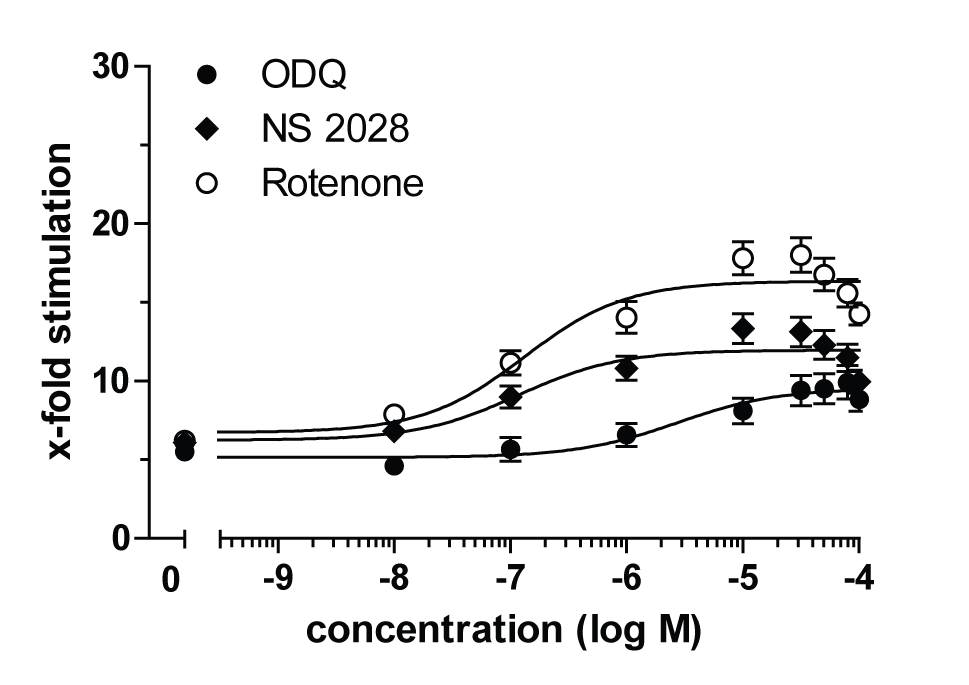

Supplement: Figure S3 — Effects of NS 2028, rotenone and ODQ on BAY 58-2667-induced TC4-WT sGC activity. cGMP reporter cells were transiently cotransfected with WT α1 sGC and TC4-WT β1 sGC and incubated with 100 nM BAY 58-2667 and increasing concentrations of NS 2028, rotenone and ODQ. sGC activity is represented as x-fold stimulation compared to transfected but non-stimulated control. Data are means ± S.E.M. from 7–18 independent experiments, performed in duplicate. Basal sGC activity resulted in 11218 RLUs. (TIF) [file pone.0023596.s003.tif]

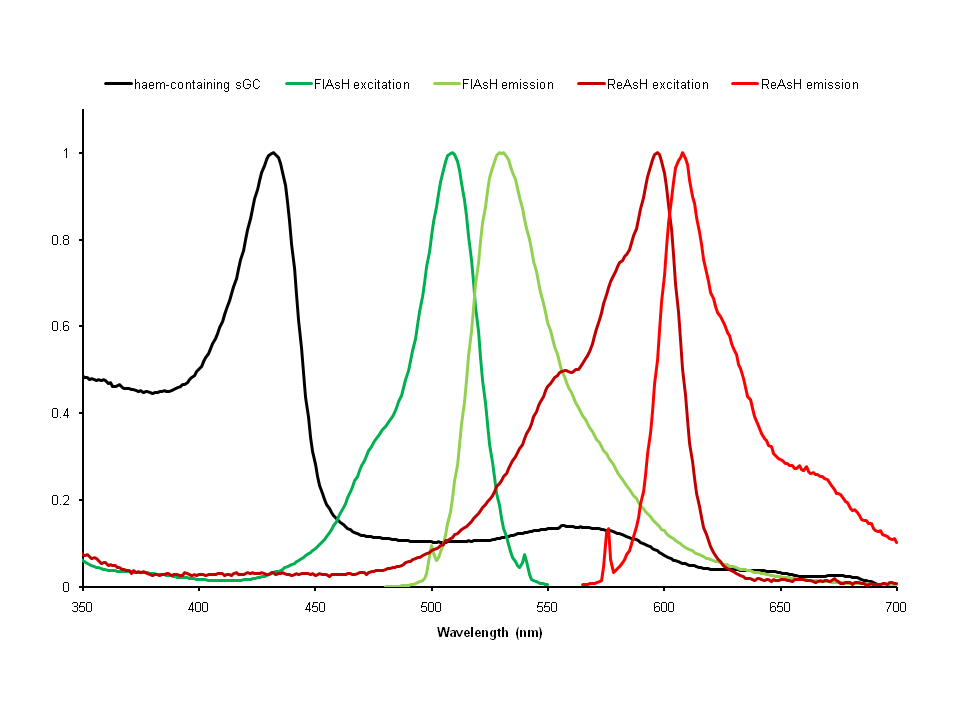

Supplement: Figure S4 — Spectra of the biarsenical dyes FlAsH and ReAsH and of Fe2+-haem-containing sGC. Data for relative fluorescence intensity of FlAsH and ReAsh was adopted from the website of Roger Tsien's lab (www.tsienlab.ucsd.edu/Documents). Absorbance spectrum of reduced sGC was adopted from [9]. (TIF) [file pone.0023596.s004.tif]

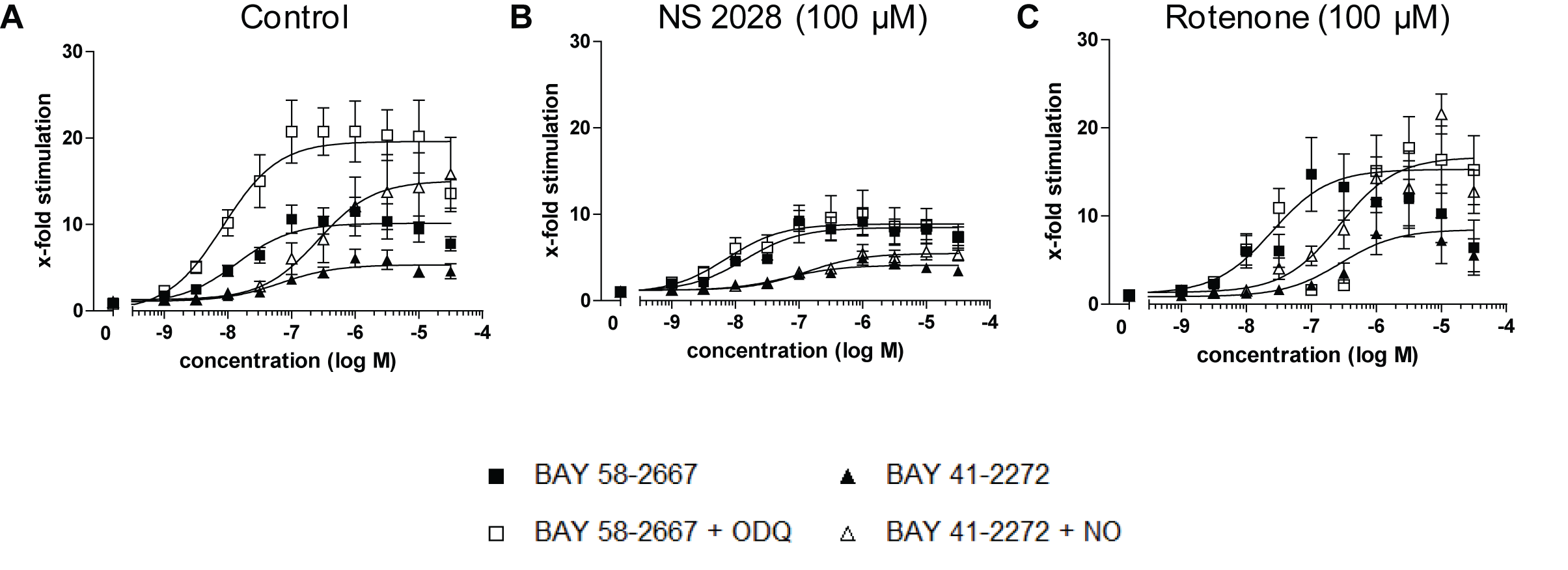

Supplement: Figure S5 — Activation pattern of WT sGC expressing cGMP reporter cells. Cells were incubated with increasing concentrations of BAY 58-2667, BAY 41-2272 alone or in combination with 10 µM ODQ or 10 nM DEA/NO (NO), respectively. (TIF) [file pone.0023596.s005.tif]
