# Supplementary material for: Fluorescence Dequenching Makes Haem-Free Soluble Guanylate Cyclase Detectable in Living Cells
Source: PLoS One. 2011 Aug 17;6(8):e23596. doi: 10.1371/journal.pone.0023596 (PMC3157391; doi:10.1371/journal.pone.0023596)
Supplement: Table S1 — Effects of 90 min pre-treatment with 100 µM NS 2028 or rotenone on WT sGC protein levels. cGMP reporter cells were transiently cotransfected with the WT α1 and β1 subunit of sGC. α1 sGC and WT β1 sGC were detected separately and protein levels were measured by densitometric measurement. sGC protein levels were normalized to the respective control which was set as 100%. Data are means ± S.E.M. from 4–6 independent experiments. **p<0.01; ***p<0.005: Student's t-test. (DOC) [file pone.0023596.s006.doc]

|  | **α_1_ sGC** | **β_1_ sGC** |
| --- | --- | --- |
| Control | 100 | 100 |
| 100 µM NS 2028 | 76.86 ± 17.52** | 83.05 ± 12.45** |
| 100 µM Rotenone | 97.45 ± 6.91 | 69.20 ± 22.75*** |
