# Supplementary material for: Fluorescence Dequenching Makes Haem-Free Soluble Guanylate Cyclase Detectable in Living Cells
Source: PLoS One. 2011 Aug 17;6(8):e23596. doi: 10.1371/journal.pone.0023596 (PMC3157391; doi:10.1371/journal.pone.0023596)
Supplement: Table S2 — Primers used to insert the tetracysteine motif or the Y135A/R139A mutation at the desired positions. (DOC) [file pone.0023596.s007.doc]

| TC aa243-248  β_1_L243C  β_1_Q244G  β_1_K247C | 5’-cagagtgctcccccagtgccagcctgggaagtgcagccttctgtctgtc-3’  5’-cagagtgctcccccagtgcggccctgggaagtgcagccttctgtctgtc-3’  5’-cagagtgctcccccagtgcggccctgggtgctgcagccttctgtctgtc-3’ |
| --- | --- |
| TC aa239-244  (TC4)  β_1_V239C  β_1_L240C  β_1_Q242G  β_1_L243C  β_1_Q244C | 5’-gaaatgctatctacagatgcctcccccagctccagcctgggaagtgcagcc-3’  5’-ggaaatgctatctacagatgctgcccccagctccagcctgggaagtgcagcc-3’  5’-ggaaatgctatctacagatgctgccccgggctccagcctgggaagtgcagcc-3’  5’-ggaaatgctatctacagatgctgccccgggtgccagcctgggaagtgcagcc-3’  5’-ggaaatgctatctacagatgctgccccgggtgctgccctgggaagtgcagcc-3’ |
| TC aa257-262  β_1_V257C  β_1_R258C  β_1_H260G  β_1_I261C  β_1_D262C | 5’-ctgtctgtcttctctctgtgccgccctcatattgacatcagtttccacggg-3’  5’-ctgtctgtcttctctctgtgctgccctcatattgacatcagtttccacggg-3’  5’-ctgtctgtcttctctctgtgctgccctggtattgacatcagtttccacggg-3’  5’-ctgtctgtcttctctctgtgctgccctggttgcgacatcagtttccacggg-3’  5’-ctgtctgtcttctctctgtgctgccctggttgctgcatcagtttccacggg-3’ |
| TC aa170-175  β_1_R170C  β_1_S171C  β_1_E172P  β_1_E173G  β_1_E175C | 5’-ggttattcagcaatgtagtgaagaatgtgatcatacccaatttttaattg-3’  5’-ggttattcagcaatgttgtgaagaatgtgatcatacccaatttttaattg-3’  5’-ggttattcagcaatgttgtccagaatgtgatcatacccaatttttaattg-3’  5’-ggttattcagcaatgttgtccaggatgtgatcatacccaatttttaattg-3’  5’-ggttattcagcaatgttgtccaggatgttgtcatacccaatttttaattg-3’ |
| TC aa111-116 | 5‘-gaccacctcgccacctgctgcccagggtgctgcgcaccttccttccggtg-3‘ |
| TC C-tag  Additional M  β_1_C-6  β_1_C-5  β_1_G-3  β_1_C-2  β_1_C-1 | 5’-cgaaattaatacgactcatgatagggagacccaagcttggtacc-3’  5’-cgactcatgatatgtagacccaagcttggtaccgagctc-3’  5’-ctcatgatatgttgtcccaagcttggtaccgagctc-3’  5’-ctcatgatatgttgtcccgggcttggtaccgagctc-3’  5’-ctcatgatatgttgtcccgggtgtggtaccgagctcggatc-3’  5’-tcatgatatgttgtcccgggtgttgtaccgagctcggatc-3’ |
| TC4-Y135A/R139A | 5’-gcaaagggctcattctgcacgcctactcggaagcagaggggc-3’ |
